# Peripheral CLOCK Regulates Target-Tissue Glucocorticoid Receptor Transcriptional Activity in a Circadian Fashion in Man

**DOI:** 10.1371/journal.pone.0025612

**Published:** 2011-09-28

**Authors:** Evangelia Charmandari, George P. Chrousos, George I. Lambrou, Aikaterini Pavlaki, Hisashi Koide, Sinnie Sin Man Ng, Tomoshige Kino

**Affiliations:** 1 First Department of Pediatrics, University of Athens Medical School, “Aghia Sophia” Children's Hospital, Athens, Greece; 2 Division of Endocrinology and Metabolism, Biomedical Research Foundation of the Academy of Athens, Athens, Greece; 3 Program in Reproductive and Adult Endocrinology, Eunice Kennedy Shriver National Institute of Child Health and Human Development, National Institutes of Health, Bethesda, Maryland, United States of America; 4 Unit on Molecular Hormone Action, Program in Reproductive and Adult Endocrinology, Eunice Kennedy Shriver National Institute of Child Health and Human Development, National Institutes of Health, Bethesda, Maryland, United States of America; 5 School of Biomedical Science, Faculty of Medicine, Chinese University of Hong Kong, Shatin, Hong Kong Special Administrative Region, China; Roswell Park Cancer Institute, United States of America

## Abstract

**Context and Objective:**

Circulating cortisol fluctuates diurnally under the control of the “master” circadian CLOCK, while the peripheral “slave” counterpart of the latter regulates the transcriptional activity of the glucocorticoid receptor (GR) at local glucocorticoid target tissues through acetylation. In this manuscript, we studied the effect of CLOCK-mediated GR acetylation on the sensitivity of peripheral tissues to glucocorticoids in humans.

**Design and Participants:**

We examined GR acetylation and mRNA expression of GR, CLOCK-related and glucocorticoid-responsive genes in peripheral blood mononuclear cells (PBMCs) obtained at 8 am and 8 pm from 10 healthy subjects, as well as in PBMCs obtained in the morning and cultured for 24 hours with exposure to 3-hour hydrocortisone pulses every 6 hours. We used EBV-transformed lymphocytes (EBVLs) as non-synchronized controls.

**Results:**

GR acetylation was higher in the morning than in the evening in PBMCs, mirroring the fluctuations of circulating cortisol in reverse phase. All known glucocorticoid-responsive genes tested responded as expected to hydrocortisone in non-synchronized EBVLs, however, some of these genes did not show the expected diurnal mRNA fluctuations in PBMCs *in vivo*. Instead, their mRNA oscillated in a Clock- and a GR acetylation-dependent fashion in naturally synchronized PBMCs cultured *ex vivo* in the absence of the endogenous glucocorticoid, suggesting that circulating cortisol might prevent circadian GR acetylation-dependent effects in some glucocorticoid-responsive genes *in vivo*.

**Conclusions:**

Peripheral CLOCK-mediated circadian acetylation of the human GR may function as a target-tissue, gene-specific counter regulatory mechanism to the actions of diurnally fluctuating cortisol, effectively decreasing tissue sensitivity to glucocorticoids in the morning and increasing it at night.

## Introduction

Human beings live under the strong influence of light/dark cycles associated with the day/night changes created by the 24-hour rotation of the earth [1]. To be acclimatized to such environmental changes, they have a highly conserved, ubiquitous molecular “clock”, the CLOCK system, which creates internal circadian rhythmicity under the influence of light/dark information and synchronizes their physical activities, such as motivational behaviors, food intake, energy metabolism, rest and sleep [1], [2], [3]. The circadian CLOCK system consists of central and peripheral components, which are located respectively in the suprachiasmatic nucleus (SCN) of the hypothalamus and virtually all remaining organs and tissues [1]. The SCN acts as a “master” CLOCK under the strong influence of light/dark input from the eyes, whereas the peripheral CLOCK behaves as a “slave”, subjugated by the former through as yet unclear mechanisms [4]. Both master and slave CLOCKs share almost the same transcriptional regulatory machinery with coordinated activation/inactivation of a set of transcription factors, including the “circadian locomotor output cycle kaput” (Clock), its heterodimer partner “brain-muscle-arnt-like protein 1” (Bmal1) and other essential negative regulators, such as the “Periods” (Pers), “Cryptochromes” (Crys), and the nuclear hormone receptors REV-erbs and retinoic acid receptor-related orphan receptors (RORs) [1]. These transcription factors create a negative feedback transcriptional loop through mutual transcriptional activation and repression that ultimately maintains an approximately 24-hour oscillation of their gene expression [1].

In addition to the predictably regular day/night changes in the environment, humans face frequent unforeseen short- and long-term influences, the “stressors” [5], [6]. To adapt to these stressful stimuli, they possess another regulatory system, the “Stress System” [5], [6]. The hypothalamic-pituitary-adrenal (HPA) axis, one of the two major arms of this system, consists of the hypothalamic paraventricular nucleus (PVN), the pituitary corticotrophs and the adrenal glands, which, respectively, employ corticotropin-releasing hormone/arginine vasopressin, adrenocorticotropic hormone (ACTH) and glucocorticoids as their signaling effector molecules [5], [6]. The human glucocorticoid cortisol secreted from the adrenal cortices circulates systemically and acts as the end-effector molecule of the HPA axis in almost all tissues and organs; its strong, pleiotropic effects are mediated by the ubiquitously expressed glucocorticoid receptor (GR) [7], [8]. Following binding to cortisol, GR regulates positively and negatively the transcriptional activity of thousands of glucocorticoid-responsive genes either by binding to glucocorticoid response elements (GREs) located in the promoter region of these genes or by physically interacting with other transcriptional factors, altering the activities of the latter on their own responsive genes [7], [8]. As glucocorticoids are involved in human physiology and pathology in a major fashion and are essential for life in primates, how their actions are mediated and modulated at the level of target tissues is a pivotal question.

Both the circadian CLOCK system and the stress-responsive HPA axis are fundamental for survival and appear to interact with each other at multiple levels [9]. For example, the master CLOCK located in the hypothalamic SCN creates the strong diurnal circadian rhythm of circulating ACTH and cortisol [9]. On the other hand, we recently found in an *in vitro* cellular system that the peripheral CLOCK negatively regulates the transcriptional activity of the GR through physical interaction with it and subsequent acetylation of multiple lysine residues (“lysine cluster”) located in its hinge region [10]. We hypothesized that this enzymatic modification of the GR acts possibly as a local, target tissue counter regulatory mechanism to the actions of the diurnally fluctuating circulating cortisol [10].

To further examine the physiologic interaction of the circadian CLOCK system and the HPA axis at peripheral glucocorticoid target tissues in humans, we performed an *in vivo* clinical study in which we examined the acetylation of the GR, as well as the mRNA expression of CLOCK-related and glucocorticoid-responsive genes employing peripheral blood mononuclear cells (PBMCs) from healthy adult subjects. Due to the marked changes that have been taking place in human lifestyle in the modern era, including a major extension of the day period, frequent trans-time-zone travel and nightshift work, investigations of the coupling of and the physiologic interactions between the circadian CLOCK system and the stress-responsive HPA axis are critical for understanding their influences on human well being and disease [9].

## Methods

### Subjects enrolled and study design

We enrolled 10 healthy subjects (3 males, 7 females, age 33.3±1.9 yr [mean ± S.E.]). Their clinical characteristics and the biochemical and endocrine parameters are summarized in Table 1. The study was approved by the “Aghia Sophia” Children's Hospital Committee on the Ethics of Human Research and written informed consent was obtained in all cases. These healthy volunteers were admitted to the Endocrine Unit on the day of the study and anthropometrics were obtained by a single trained observer. Blood samples for biochemical and endocrine investigations, as well as for purification of PBMCs were drawn twice, at 8 am, following a 12-hour overnight fast, and at 8 pm of the same day. They were instructed to have regular meals in the day of testing after an overnight fast. Serological tests for plasma fasting glucose, serum cholesterol and triglyceride levels, and white blood cell counts were performed in the Clinical Chemistry Laboratory of the “Aghia Sophia” Children's Hospital.

**Table 1:** Clinical characteristics and endocrine parameters of the subjects enrolled in the study.

Parameters (units)	Subjects (Mean ± S.E.)	Normal range (Mean ± S.E.)
Sex	Male: 3, Female: 7	N/A
Age (yr)	33.3±1.84	N/A
Body mass index (kg/m^2^)	25.07±1.43	18.5–25
White blood cell number (×10^3^/µL)	7.42±0.66	3.50–11.00
% of lymphocytes (%)	27±2	16.25–45.00
Fasting plasma glucose (mg/dL)	93.70±3.52	70–110
Total cholesterol (mg/dL)	197.30±11.73	120–200
Triglycerides (mg/dL)	95.90±17.79	30–130
Serum cortisol at 8 am (nM)	51.3±5.52	12.0–53.6
Serum cortisol at 8 pm (nM)	13.9±4.39	12.0–53.6
Plasma ACTH at 8 am (pM)	0.057±0.008	0.015–0.139
Plasma ACTH at 8 pm (pM)	0.022±0.007	0.015–0.139

ACTH: adrenocorticotropic hormone.

We also obtained at 6 am PBMCs from 6 additional healthy subjects (3 males, 3 females, 35.6±1.68 yr [mean ± S.E.]) to perform an *ex vivo* examination of GR acetylation and circadian mRNA expression of selected CLOCK-related and glucocorticoid-responsive genes.

### Purification of PBMCs from whole blood and establishment of Epstein-Barr virus-transformed peripheral lymphocytes

PBMCs were purified from whole blood by using Ficoll-Paque PLUS (GE Healthcare Biosciences, Piscataway, NJ). As non-synchronized control cells, we employed Epstein-Barr virus (EBV)-transformed peripheral lymphocytes (B lymphoblasts) that were established from PBMCs as previously described [11]. By measuring mRNA expression of CLOCK-related genes, we found, as expected, that the circadian rhythm of EBV-transformed peripheral lymphocytes were not synchronized due to long maintenance in culture media (data not shown).

### Knockdown of CLOCK mRNA in PBMCs cultured *ex vivo*

PBMCs obtained at 6 am were transfected with Clock or control siRNA (Santa Cruz Biotechnologies, Inc., Santa Cruz, CA) by using the Nucleofector system (Lonza Group Ltd., Basel, Switzerland) and the AMAXA® Human T Cell Nucleofector Kit (Lonza Group Ltd.) with over 80% transfection efficiency and cell viability, as previously described [12].

### Total RNA isolation and SYBR Green real-time PCR

Total RNA was purified from PBMCs or EBV-transformed peripheral lymphocytes by using Trizol® Reagent (Invitrogen), treated with DNase I (Promega, Madison, MI), and were subsequently reverse transcribed to cDNA with TaqMan reverse transcription reagents (Applied Biosystems, Carlsbad, CA). The SYBR Green-based real-time PCR was performed as previously described [10]. The primer pairs used for measuring mRNA levels of the GR, CLOCK-related and glucocorticoid-responsive proteins are shown in Table 2. The obtained C*t* (threshold cycle) values of these mRNAs were normalized for mean C*t* values of the β-actin, glyceraldehyde-3-phosphate dehydrogenase (GAPDH) and ribosomal protein large P0 (RPLP0) mRNAs, and their relative expressions were shown as fold induction over the mean values of all subjects. The dissociation curves of primer pairs used showed a single peak and samples after PCR reactions had a single expected DNA band in an agarose gel analysis (data not shown).

**Table 2:** Primer pairs used in real-time PCR mRNA quantitations.

Gene name		Primer sequence
*GRα*	Forward	5′-GTCAAGAGGGAAGGAAACTC-3′
	Reverse	5′-CAATACTCATGGTCTTATCC-3′
CLOCK-related genes		
*Bmal1*	Forward	5′-CTAAGGATGGCTGTTCAG-3′
	Reverse	5′-CTGCTGCCCTGAGAATGAG-3′
*Clock*	Forward	5′-GAAGTTAGGGCTGAAAGAC-3′
	Reverse	5′-GATCAAACCTTTCCAATGC-3′
*Cry1*	Forward	5′-CAACCAGCAGATGTGTTTCC-3′
	Reverse	5′-GACTTCTACTCCAGCTTCAG-3′
*RORα*	Forward	5′-GTCGATTACAGAAATGC-3′
	Reverse	5′-CTGCATCCGGTGTTTCTG-3′
*Per1*	Forward	5′-GATGTGGGAGTCTTCTATGG-3′
	Reverse	5′-CAGGACCTCCTCTGATACG-3′
Genes up-regulated by glucocorticoids		
*Annexin A1*	Forward	5′-GTCGCTGCCTTGCATAAGG-3′
	Reverse	5′-GTTTCATCCAGGGGCTTTCC-3′
*DUSP1*	Forward	5′-CAAGTCTTCTTCCTCAAAGG-3′
	Reverse	5′-GAACTGCACCCAGATTCC-3′
*GILZ*	Forward	5′-GATGTGGTTTCCGTTAAGC-3′
	Reverse	5′-CTCTCTCACAGCATACATCAG-3′
*Tristetraprolin*	Forward	5′-CATGGCCAACCGTTACAC-3′
	Reverse	5′-GTCCCTCCATGGTCGGATGG-3′
Genes down-regulated by glucocorticoids		
*IFNγ*	Forward	5′-CAGGACCCATATGTAAAAGAAG-3′
	Reverse	5′-CTGTCACTCTCCTCTTTCC-3′
*IL-1α*	Forward	5′-GACCTGAAGAACTGTTACAG-3′
	Reverse	5′-GATCCATGCAGCCTTCATG-3′
*IL-12 p40*	Forward	5′-GAGGTTCTAAGCCATTCG-3′
	Reverse	5′-CCACCAGCAGGTGAAACG-3′
*TNFα*	Forward	5′-CTGCCTGCTGCACTTTGG-3′
	Reverse	5′-CTCAGCTTGAGGGTTTGC-3′
Control genes		
*β-Actin*	Forward	5′-CAACCGCGAGAAGATGAC-3′
	Reverse	5′-GTCACCGGAGTCCATCAC-3′
*GAPDH*	Forward	5′-CAATGACCCCTTCATTGAC-3′
	Reverse	5′-GATGGTGATGGGATTTCC-3′
*RPLP0*	Forward	5′-CCAGCTCTGGAGAAACTG-3′
	Reverse	5′-CTTCACATGGGGCAATGG-3′

Bmal1: brain-muscle-arnt-like protein 1, Clock: circadian locomotor output cycle kaput, Cry1: *Cryptochrome 1*, DUSP1: dual specificity phosphatase 1, GAPDH: glyceraldehyde-3-phosphate dehydrogenase, GILZ: glucocorticoid-responsive leucine zipper protein, GRα: glucocorticoid receptor α, IFNγ: interferon γ, IL-1α: interleukin-1α, Per1: period 1, RORα: retinoic acid receptor-related orphan receptor α, RPLP0: ribosomal protein large P0.

### Diurnal and hydrocortisone-induced mRNA expressions in PBMCs and EBV-transformed peripheral lymphocytes

The mRNA levels of the *GR*, CLOCK-related and glucocorticoid-responsive genes were evaluated in PBMCs obtained at 8 am and 8 pm from 10 healthy subjects by performing SYBR Green real-time PCR. PBMCs obtained at 6 am from 6 healthy subjects were dispersed in RPMI 1640 medium supplemented with 10% charcoal/dextran-treated fetal bovine serum (FBS) (Thermo Fisher Scientific, Inc., Waltham, MA), 100 U/ml of penicillin and 100 µg/ml of streptomycin at a density of 1×10^7^ cells/ml. They were incubated with 5×10^−7^ M of hydrocortisone (Sigma-Aldrich, St. Louis, MO) or the vehicle ethanol for 3 hours at every 6 hours.

The EBV-transformed peripheral lymphocytes were also maintained in the same medium at a density of 5×10^6^ cells/ml, and were incubated with 5×10^−7^ M of hydrocortisone or the vehicle ethanol for 5 hours. The mRNA expressions of *GR*, CLOCK-related and glucocorticoid-responsive genes were determined in the SYBR Green real-time PCR. Hydrocortisone-induced fold mRNA expression was calculated by dividing the values obtained in the presence of hydrocortisone by those obtained in its absence (values obtained in the absence of hydrocortisone are shown as “1”).

### Evaluation of GR acetylation in PBMCs

PBMCs from 5 subjects purified from blood samples drawn at 8 am and at 8 pm or PBMCs cultured *ex vivo* in RPMI 1640 medium supplemented with 10% charcoal/dextran-treated FBS, 100 U/ml of penicillin and 100 µg/ml of streptomycin at a density of 1×10^7^ cells/ml were lysed in buffer containing 50 mM Tris-HCl [pH 7.4], 150 mM NaCl, 0.1% SDS, 1% NP-40, 0.5% sodium deoxycholate and 1Tab/50 ml Complete™ Tablet (Roche Applied Science, Indianapolis, IN), and whole homogenates were obtained by centrifugation. Immunoprecipitation of GR was carried out by using anti-GRα antibody (Santa Cruz Biotechnology, Inc., Santa Cruz, CA), as previously described [10]. GR was purified in 10% NuPAGE® Novex® Bis-Tris gels (Invitrogen, Carlsbad, CA), blotted on nitrocellulose membranes, and whole and acetylated GR was detected by anti-GRα (Santa Cruz Biotechnology, Inc.) and anti-acetylated lysine (Millipore, Billerica, MA, USA) antibody, respectively. Band intensities of the acetylated GR measured with the ImageJ 1.43u software (National Institutes of Health, Bethesda, MD, USA) were normalized for those of whole GR, and relative acetylation of GR was expressed as fold acetylation against the control indicated.

### Statistical analysis

Statistical analysis was performed by using the Mann-Whitney test for the serum measurements, mRNA expression of the genes indicated and GR acetylation of samples obtained at 8 am and at 8 pm, while an unpaired Student t-test with two-tailed p values was employed for those obtained from PBMCs or EBV-transformed peripheral lymphocytes cultured in the presence or absence of hydrocortisone. We used “n” for indicating the number of subjects tested and “m” for the number of measurements performed.

## Results

**Figure 1:**
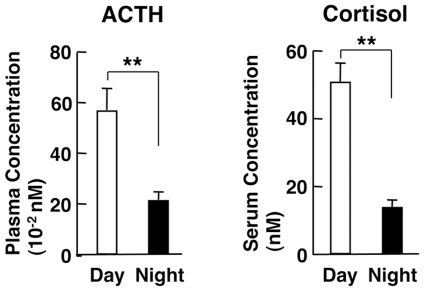
Concentrations of plasma ACTH and serum cortisol at 8 am and 8 pm in healthy adult volunteers. Concentrations of plasma ACTH (left panel) and serum cortisol (right panel) at 8 am (Day) and 8 pm (Night) are shown. Bars represent the mean ± S.E. values of serum cortisol and plasma ACTH. **: P<0.01, compared to the conditions indicated (n = 10, m = 10).

We first measured plasma ACTH and serum cortisol concentrations in 10 subjects enrolled in this study (Table 1) for evaluation of the circadian regulation of their HPA axis at 8 am and 8 pm within the same day. As expected, values of these two parameters were ∼2–4 fold higher in the morning than in the evening, indicating that the activity of their HPA axis fluctuates in a circadian fashion, consistent with previous reports [13] (Figure 1).

**Figure 2:**
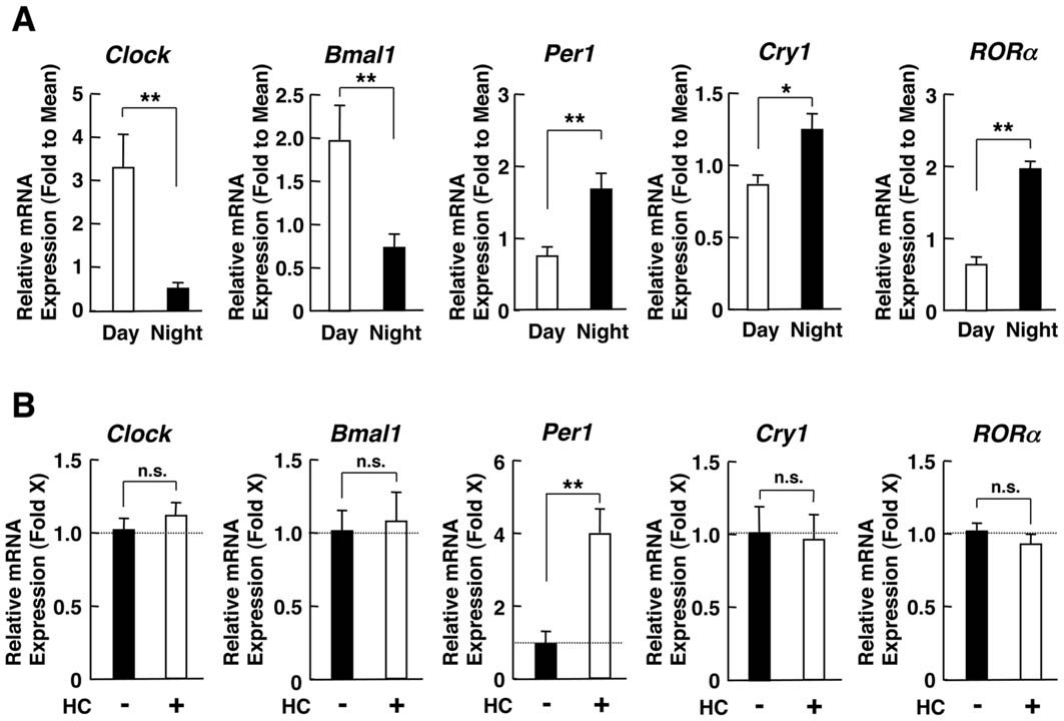
Daily change of CLOCK-related gene mRNA expressions in PBMCs (A) and their response to hydrocortisone in EBV-transformed peripheral lymphocytes (B). **A:** Morning and evening mRNA expression of CLOCK-related genes in PBMCs. Relative mRNA expression of *Clock*, *Bmal1*, *Per1*, *Cry1* and *RORα* at 8 am (Day) and 8 pm (Night) in PMBCs obtained from 10 healthy subjects is shown. The measurement was performed in duplicate for each subject. Bars represent mean ± S.E. values of relative mRNA expression of the genes indicated. **: P<0.01, compared to the conditions indicated (n = 10, m = 20). **B:** The effect of hydrocortisone on the mRNA expression of CLOCK-related genes in EBV-transformed peripheral lymphocytes. EBV-transformed peripheral lymphocytes were incubated with 5×10^−7^ M of hydrocortisone (HC) for 5 hours and mRNA expression of CLOCK-related genes was evaluated. The measurement was performed in triplicate. Bars represent mean ± S.E. values of hydrocortisone (HC)-induced fold mRNA expression of indicated genes. **: P<0.01, compared to the conditions indicated (m = 3).

We next examined the mRNA expression of the CLOCK-related genes *Clock*, *Bmal1*, *Per1*, *Cry1*, and *RORα* in PBMCs from the same subjects obtained at 8 am and at 8 pm (Figure 2A). *Clock* and *Bmal1* mRNA expression was higher in the samples obtained at 8 am than in those collected at 8 pm, suggesting that they are under circadian regulation in PBMCs. In contrast to these molecules, the mRNA expression of *Per1* and *Cry1* were higher at night than in the morning, further indicating that they were under reciprocal circadian regulation opposed to that of Clock/Bmal1. The mRNA expression of *RORα*, a component of the auxiliary loop of the circadian CLOCK transcriptional system, also demonstrated the same circadian rhythmicity as *Per1* and *Cry1*, with higher mRNA expression levels in the evening than in the morning. The circadian rhythm of the CLOCK-related genes observed in our subjects was overall consistent with previously reported findings obtained in human PBMCs and dermal fibroblasts [14], [15], [16].

We also tested the responsiveness of these CLOCK-related genes to hydrocortisone (cortisol) by treating EBV-transformed lymphocytes with 5×10^−7^ M of this glucocorticoid for 5 hours. As expected, these cells were not synchronized by their CLOCK circadian system as explained in Materials and Methods. The concentration of hydrocortisone employed is equivalent to the peak value of cortisol in serum [17]. We found that the mRNA expressions of all the CLOCK-related genes, except *Per1*, were not responsive to hydrocortisone (Figure 2B), suggesting that the daily fluctuations seen in our subjects were under their own intrinsic regulation, independent of the changes observed in circulating cortisol concentrations.

**Figure 3:**
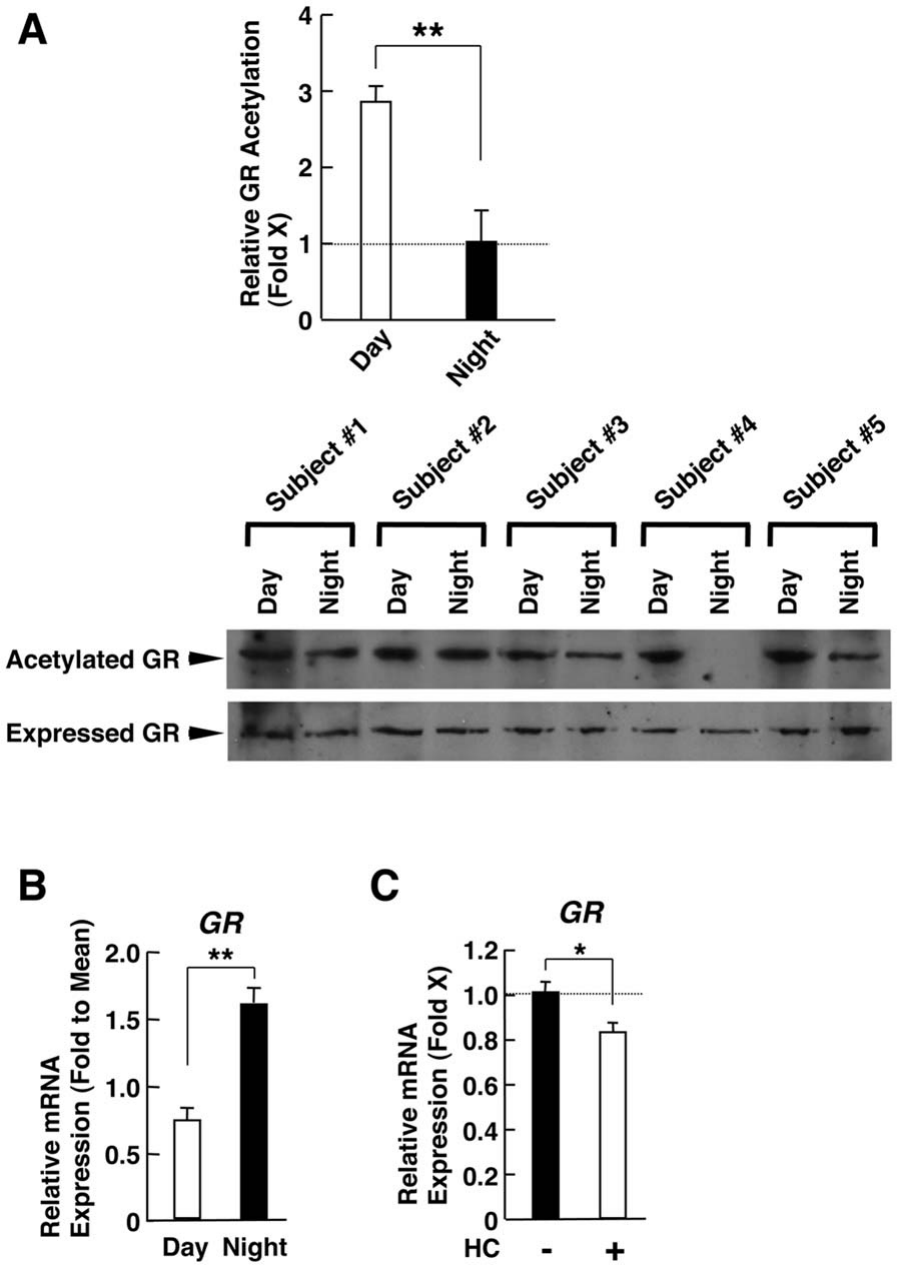
Daily changes of the GR acetylation and its mRNA expression in PBMCs, and response of *GR* mRNA expression to hydrocortisone in EBV-transformed peripheral lymphocytes. **A:** Acetylation of GR protein is high in the morning and low in the evening. Acetylation of GR was examined in whole cell lysates obtained at 8 am (Day) and 8 pm (Night) from PBMCs purified from 5 subjects. Band intensity of acetylated GR obtained in 3 independent experiments was corrected for that of total precipitated GR, and relative GR acetylation was calculated as the mean value of all subjects' measurements corrected values at Night as “1”. Bars in the top panel represent mean ± S.E. values of relative GR acetylation, while representative Western blot images are shown in the bottom panel. **: P<0.01, compared to conditions indicated (n = 5, m = 15). **B and C:** Daily change of the *GR* mRNA expression in PBMCs (**B**) and its response to hydrocortisone in EBV-transformed peripheral lymphocytes (**C**). Relative *GR* mRNA expression at 8 am (Day) and at 8 pm (Night) in PBMCs (**B**) and the effect of hydrocortisone (HC) on GR mRNA expression in EBV-transformed peripheral lymphocytes (**C**) are shown. EBV-transformed peripheral lymphocytes were incubated with 5×10^−7^ M of hydrocortisone (HC) for 5 hours. The measurements were performed in duplicate and in triplicate for panel B and C, respectively. Bars represent mean ± S.E. values of relative GR mRNA expression (**B**) or hydrocortisone (HC)-induced fold GR mRNA expression (**C**). *: P<0.05, **: P<0.01, compared to the conditions indicated (**B**: n = 10, m = 20, **C**: m = 3).

We then examined acetylation of the GR in PBMCs from 5 of the 10 subjects enrolled in this study obtained at 8 am and 8 pm (Figure 3A). In these subjects, the circadian changes of the circulating concentrations of ACTH and cortisol were similar to those of the 5 subjects who only had the hormonal measurements (p = 0.63 and 0.73 for ACTH and cortisol, respectively). We normalized the amounts of acetylated GR for the amounts of total precipitated GR in PBMCs. The calculated mean ± SE ratio was approximately 2.8±0.44-fold higher in the morning than in the evening, suggesting that Clock/Baml1 acetylates GR in a circadian fashion in these cells (p<0.01).

We then examined the mRNA expression of the *GR* in PBMCs (Figure 3B and 3C). We found that *GR* mRNA expression fluctuated in these cells with ∼2-fold higher values in the evening than in the morning (Figure 3B). Given that the *GR* mRNA expression was weakly suppressed by hydrocortisone treatment in EBV-transformed peripheral lymphocytes (Figure 3C), the daily fluctuation of *GR* mRNA may in part be in response to the oscillation of circulating cortisol concentrations (homologous hormone-receptor downregulation).

**Figure 4:**
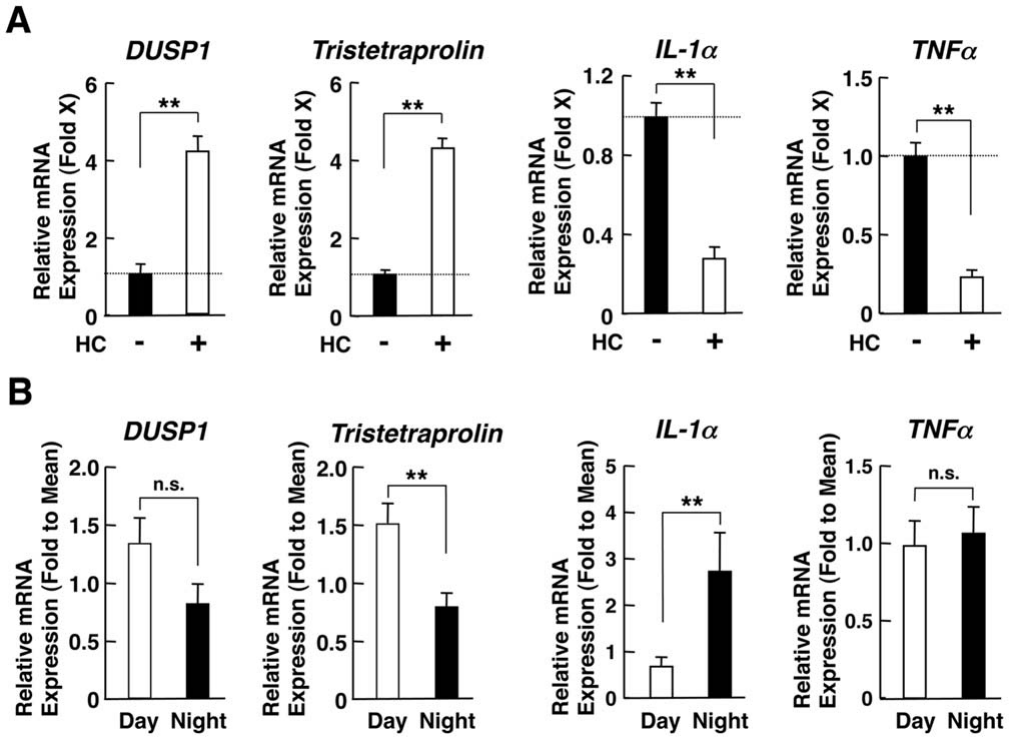
Response of glucocorticoid-responsive gene mRNA expressions to hydrocortisone in EBV-transformed peripheral lymphocytes and their daily changes in PBMCs. **A:** The effect of hydrocortisone on the expression of the mRNAs of known glucocorticoid-responsive genes in EBV-transformed peripheral lymphocytes. Samples obtained as in Figure 3 were used for the evaluation of mRNA expression of the known glucocorticoid-responsive genes indicated. *DUSP1* and *tristetraprolin* are the genes known to be up-regulated by glucocorticoids, while *IL-1α* and *TNFα* represent those known to be down-regulated. The measurements were performed in triplicate. Bars represent the mean ± S.E. values of hydrocortisone (HC)-induced fold mRNA expression of indicated genes. **: P<0.01, compared to the conditions indicated (m = 3). **B:** mRNA expression of the known glucocorticoid-responsive genes in the morning and in the evening. Relative mRNA expression of *DUSP1, tristetraprolin, IL-1α* and *TNFα* at 8 am (Day) and at 8 pm (Night) in PMBCs obtained from 10 healthy subjects is shown. The measurements were performed in duplicate for each subject. Bars represent mean ± S.E. values of relative mRNA expression of the genes indicated. **: P<0.01, n.s.: not significant, compared to the conditions indicated (n = 10, m = 20).

We further examined mRNA expression of glucocorticoid-responsive genes in PBMCs to evaluate local effectiveness of circulating cortisol. We chose the annexin A1, dual specificity phosphatase 1 (DUSP1), glucocorticoid-inducible leucine zipper protein (GILZ) and the tristetraprolin as the genes up-regulated by glucocorticoids, while we measured mRNA expression of interferon γ (IFNγ), interleukin-1α (IL-1α), IL-12 p40 and tumor necrosis factor α (TNFα) as the genes down-regulated by glucocorticoids. Results of *DUSP1, tristetraprolin, IL-1α and TNFα* are shown in Figure 4, while those of *Annexin A1, GILZ, INFγ and IL-12 p40* are demonstrated in Supplemental Figure S1.

Consistently with previous reports [18], [19], the mRNA expression of these genes in EBV-transformed lymphocytes that were not in circadian synchronization responded strongly to the 5-hour treatment with 5×10^−7^ M of hydrocortisone either positively or negatively (Figure 4A and Supplemental Figure S1A). Time-course analysis for mRNA expression of representative *DUSP1* and *TNFα* mRNAs further indicated that these genes responded strongly to this concentration of hydrocortisone in these cells (data not shown). Their protein expression was also induced/suppressed by this steroid in the same cells (data not shown). In PBMCs obtained at 8 am and at 8 pm, mRNA expressions of *tristetraprolin* and *GILZ* were significantly higher in the morning than in the evening, indicating that their mRNA expression fluctuated diurnally quite likely under the influence of oscillating circulating cortisol (Figure 4B and Supplemental Figure S1B). In contrast to these genes, however, the mRNA expression of *DUSP1* and *annexin A1* did not fluctuate at all, despite the fact that their mRNA expression responded well to exogenously administered hydrocortisone, similarly to *tristetraproli*n and *GILZ*. Likewise, the mRNA expression of *IL-1α*, *IFNγ* and *IL-12 p40* fluctuated in PBMCs, and was significantly higher in the evening than in the morning, possibly responding negatively to circulating cortisol, while that of *TNFα* did not show such a daily fluctuation pattern (Figure 4B and Supplemental Figure S1B). Indeed, daily changes of *IFNγ* mRNA expression appeared to be blunted compared to those of *IL-1α* and *IL-12 p40*, although all these genes responded similarly to exogenously administered hydrocortisone in EBV-transformed lymphocytes with an unsynchronized CLOCK system (Figure 4A and Supplemental Figure S1A).

**Figure 5:**
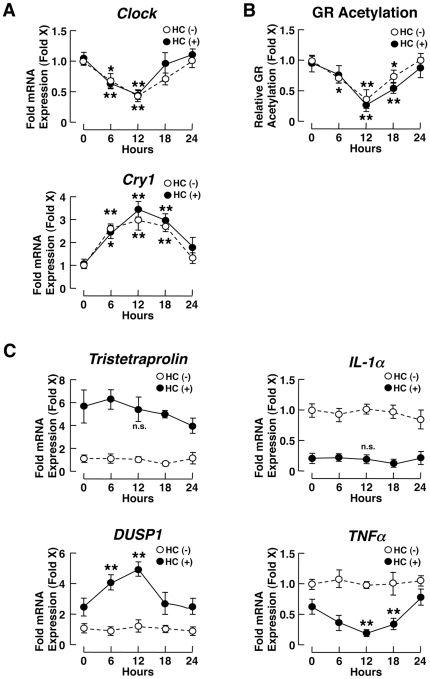
CLOCK regulates mRNA expression of glucocorticoid-responsive genes in a gene-specific fashion *ex vivo*. PBMCs obtained at 6 am from 6 healthy subjects were cultured in the medium and were treated with 5×10^−7^ M of hydrocortisone (HC) for 3 hours at every 6 hours. mRNA levels of *Clock* and *Cry1* (**A**), acetylation of the GR (**B**) and mRNA levels of glucocorticoid-responsive genes *DUSP1*, *tristetraprolin*, *IL-1α* and *TNFα* (**C**) were determined. The measurements were performed in duplicate for each subject. Circles represent the mean ± S.E. values of hydrocortisone (HC)-induced fold mRNA expression of the genes indicated and fold acetylation of GR. Values obtained in the absence of hydrocortisone (HC) at time “0” were employed as controls for glucocorticoid-responsive genes, while values obtained in the presence of hydrocortisone (HC) at time “0” were used as controls *: p<0.05, **: P<0.01, n.s.: not significant, compared to the values obtained at time “0” in the presence of hydrocortisone (n = 6, m = 12).

**Figure 6:**
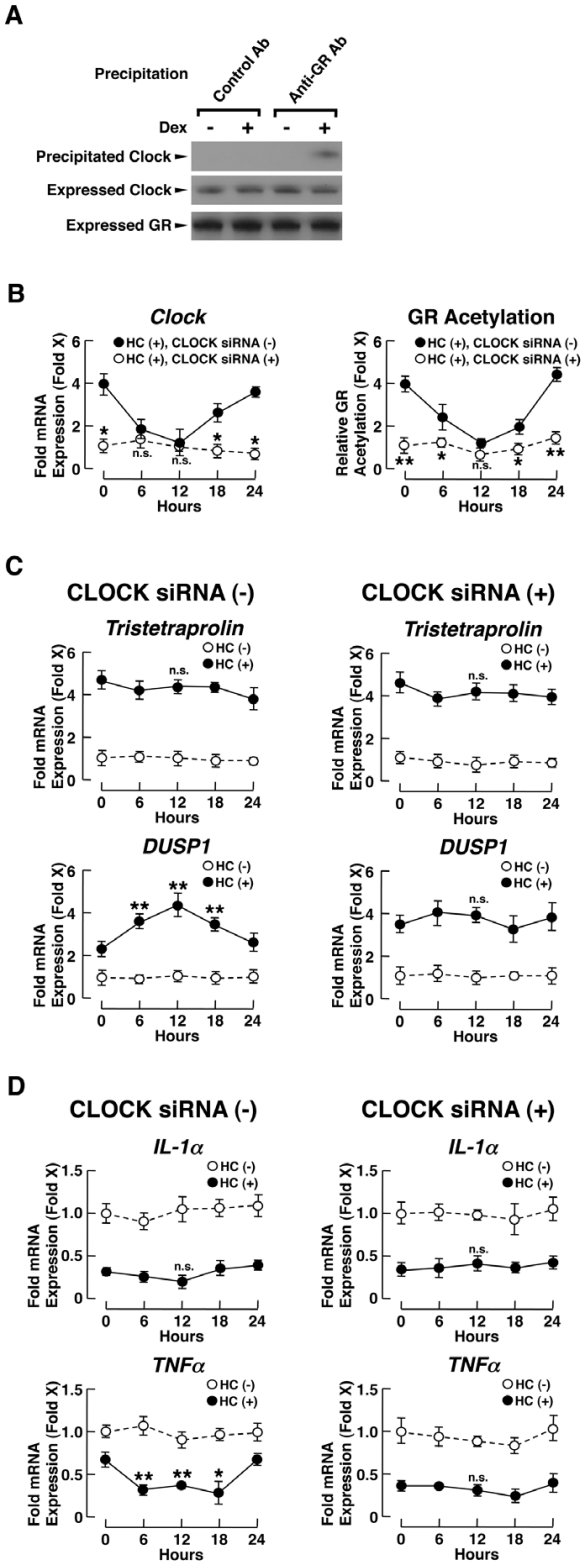
CLOCK regulates mRNA expression of glucocorticoid-responsive genes possibly through acetylation of GR by Clock. **A:** Clock and GR are associated in a hydrocortisone-dependent fashion in PBMCs cultured *ex vivo*. PBMCs obtained at 6 am from 6 healthy subjects were cultured in the absence or presence of 5×10^−7^ M of hydrocortisone (HC) for 3 hours and co-immunoprecipitation using anti-GR or control antibody was performed. Top panel indicates results of co-immunoprecipitation, while the bottom two panels show expression of Clock and GR in Western blots using their specific antibodies. Representative images of 3 independent experiments are shown. **B, C and D:** Knockdown of Clock abolishes diurnal fluctuation of *DUSP1* and *TNFα* mRNA expression in PBMCs cultured *ex vivo*. PBMCs obtained at 6 am from 3 healthy subjects were transfected with Clock or control siRNA and were treated with 5×10^−7^ M of hydrocortisone (HC) for 3 hours at every 6 hours. mRNA levels of *Clock* and acetylation of the GR (**B**), mRNA expression of glucocorticoid-responsive genes, *DUSP1*, *tristetraprolin*, *IL-1α* and *TNFα* (**C** and **D**) were determined. Experiments were performed in duplicate for each subject. Circles represent the mean ± S.E. values of fold mRNA expression of the indicated genes obtained in the absence and presence of hydrocortisone (HC). The values obtained in the absence of hydrocortisone (HC) were employed as controls. *: P<0.05, **: P<0.01, n.s.: not significant, compared to the values obtained in the absence of hydrocortisone (HC) at the same time-point for *Clock* mRNA expression and GR acetylation, and to the values obtained in the presence of hydrocortisone (HC) at time “0” for mRNA expression of glucocorticoid-responsive genes (n = 3, m = 6).

To further examine gene-specific circadian regulation of glucocorticoid-responsive genes, we purified PBMCs from 6 healthy subjects in the morning and monitored mRNA expression of *tristetraprolin*, *IL-1α*, *DUSP1*, *TNFα* and *annexin A1*, while treating these cells with 5×10^−7^ M of hydrocortisone with 3-hour pulses, every 6 hours for over a 24-hour period (Figure 5 and Supplemental Figure S2). The former 2 genes demonstrated diurnally fluctuating mRNA expression in PBMCs obtained at 8 am and 8 pm, while the latter 3 genes did not (Figure 4B and Supplemental Figure S1B). We also measured mRNA expression of *Clock* and *Cry1* and evaluated acetylation of GR at these time points. This *ex vivo* experiment gave us an opportunity to monitor the transcriptional effect of exogenously administered hydrocortisone on glucocorticoid-responsive genes in the absence of circulating cortisol, but still under the influence of the functioning CLOCK system and its circadian GR acetylation. Indeed, *Clock* and *Cry1* mRNAs demonstrated circadian fluctuation, as did acetylation of the GR (Figure 5A and 5B), in direct synchrony with *Clock* mRNA and in inverse synchrony with *Cry1* mRNA. In this culture condition, the mRNA levels of *tristetraprolin* and *IL-1α* did not fluctuate at all after treatment with hydrocortisone, while those of *DUSP1*, *TNFα* and *annexin A1* showed diurnal fluctuation, possibly due to unresponsiveness of the former genes to GR acetylation by Clock, and responsiveness of the latter genes to this GR modification (Figure 5C and Supplemental Figure S2). We further found that Clock and GR were associated with each other in a hydrocortisone-dependent fashion (Figure 6A). In agreement with these results and reasoning, knockdown of Clock by its siRNA abolished GR acetylation and the diurnal fluctuation of *DUSP1*, *TNFα* and *annexin A1* mRNA, while it did not influence mRNA expression of tristetraprolin and *IL-1α* (Figure 6B, 6C and 6D, and Supplemental Figure S3). Further, Clock knockdown did not influence mRNA expression of *DUP1* and *TNFα* in the absence of hydrocortisone (data not shown), indicating that Clock caused diurnal fluctuation of their mRNA expression through the GR, rather than influencing their expression directly.

## Discussion

We demonstrated that the mRNA levels of CLOCK-related genes fluctuated in a circadian fashion in PBMCs. Similarly, the acetylated GR was increased in the morning and decreased in the evening in these cells, possibly as a result of the diurnally oscillating Clock expression [10]. Acetylation of the GR continued to fluctuate in a circadian fashion in PBMCs cultured *ex vivo* in synchrony with Clock mRNA levels. The ratio of am to pm acetylated GR was approximately 2.8/1, which is sufficient to influence the overall activity of the GR in target tissues. Interestingly, the mRNA expression of some glucocorticoid-responsive genes had no apparent circadian regulation in PBMCs *in vivo*. The responsiveness of these glucocorticoid-responsive genes to hydrocortisone in PBMCs cultured *ex vivo,* in the absence of exposure to circulating cortisol, fluctuated diurnally, in concert with the circadian rhythmicity of GR acetylation caused by the apparently synchronized and functional CLOCK system. Taken together, these findings indicate that the transcriptional activity of the GR is generally regulated by circulating cortisol in PBMCs accompanying the latter's diurnal fluctuation, while acetylation of the GR by Clock may attenuate the transcriptional activity of the receptor stimulated by circulating cortisol in a gene- and, probably, tissue-specific fashion, functioning as a local counter regulatory mechanism(s) for the concentrations of the strongly circadian serum cortisol. Since *GR* mRNA expression in PBMCs also mirrored the fluctuation of circulating cortisol concentrations via autologous downregulation, it is possible that negative regulation functioning at the receptor concentration level might have contributed to the attenuation of GR transcriptional activity on some glucocorticoid-responsive genes in the morning and to potentiation on the same genes in the evening. We believe that the contribution of this phenomenon is limited granted that the degree of autologous glucocorticoid-induced GR downregulation is usually at the level of 10–20%, as we demonstrated in EBV-transformed lymphocytes.

We propose that gene-specific daily fluctuation of local glucocorticoid action promoted by concerted regulation of central and peripheral CLOCKs, respectively, on the circulating cortisol concentrations and on target tissue GR transcriptional activity, is essential for the maintenance of glucocorticoid action at all tissues of the human body. This hypothesis is supported by the fact that loss of this relation, as seen during chronic stress or because of excessive exogenous glucocorticoid administration with extended evening effects, lead to development of Cushing syndrome or overlapping metabolic syndrome manifestations and the resultant atherosclerosis and cardiovascular complications [9]. This is further supported by the high risk of cardiovascular manifestations often observed in people who perform day/night-shift work or are involved in frequent trans-time zone travel, possibly mediated by uncoupling of the SCN and peripheral CLOCK systems, leading to increased time-integrated exposure of target tissues to glucocorticoids [9], [20], [21]. Further research is needed to elucidate the detailed interactions between the master and slave circadian CLOCK systems and the HPA axis/glucocorticoid signaling system at local tissues in common pathological conditions that extend beyond metabolic and cardiovascular diseases to include psychiatric, inflammatory/autoimmune and sleep disorders, all known to have both circadian and stress system components [5]. It is also known that glucocorticoids influence the circadian CLOCK system by affecting expression levels of some of its components [22]. Thus, regulation appears to be mutual, adding another level of complexity. Circulating cortisol concentrations demonstrate short-term ultradian oscillation within the daily circadian oscillation [23]. It would be interesting to examine the influence of ultradian cortisol changes on Clock-induced GR acetylation-mediated regulation of peripheral GR activity in the future.

We do not know how GR acetylation regulates GR-induced transcriptional activity in a gene- or tissue-specific fashion, but it is possible that different types of GREs located in the regulatory regions of specific glucocorticoid-responsive genes interact with intact *vs.* acetylated GR with different affinities, while the negative charge and altered allosteric properties of the acetylated GR may influence its interactions with local ancillary transcription factors and specific chromatin regions of individual genes. A recent report indicates that binding of GR to GREs within different DNA sequences causes specific conformational changes in the GR ligand-binding domain (LBD) and influences the transcriptional activity of the receptor [24]. Thus, acetylation of GR at the hinge region might also influence functions of its neighboring subdomains, the DNA-binding domain and the LBD. Further, Clock acetylates GR at 4 different lysine residues located in its hinge region [10], thus it is likely that differential acetylation of one or some of these lysines could produce different effects on GR-induced transcriptional activity and this might in part explain the stochastic regulation of GR transcriptional activity in a cell [25]. In agreement with this consideration, we compared the levels of GR acetylation to mRNA expression of glucocorticoid-responsive genes, however we did not obtain conclusive results, possibly due to major variation of GR acetylation between subjects (data not shown).

We found that GR acetylation attenuated not only the transactivation but also the transrepression of glucocorticoid-responsive genes, although we previously reported that GR acetylation enhanced its suppressive effect on a synthetic nuclear factor of κB (NFκB)-responsive promoter in a reporter assay [10]. The results obtained in this study are consistent with a previous report indicating that acetylation of the human GR at lysines 494 and 495 neutralizes the negative effect of the GR on NFκB in the latter's ability to transcactivate its own responsive genes [26]. Indeed, NFκB is a strong inducer of TNFα [27], whose mRNA, however, did not fluctuate diurnally in this study. Therefore, it is possible that morning GR acetylation abolishes the circadian oscillation of some glucocorticoid-repressed genes through alterations in the transcriptional activity of NFκB. Alternatively, Clock might influence the transrepressive activity of GR by changing its interaction with recently reported ubiquitous negative GREs through which GR directly represses transcription of glucocorticoid-responsive genes [28]. The detailed mechanisms of acetylation-mediated regulation of the transrepressive effects of the GR are targets of future research.

## Supporting Information

Figure S1 Response of glucocorticoid-responsive gene mRNA expressions to hydrocortisone in EBV-transformed peripheral lymphocytes and their daily changes in PBMCs. **A:** The effect of hydrocortisone on the expression of the mRNAs of known glucocorticoid-responsive genes in EBV-transformed peripheral lymphocytes. Samples obtained as in Figure 3 were used for the evaluation of the mRNA expressions of the known glucocorticoid-responsive genes indicated. *Annexin A1* and *GILZ* are known to be up-regulated by glucocorticoids, while *IFNγ* and *IL-12 p40* are known to be down-regulated. Bars represent the mean ± S.E. values of hydrocortisone (HC)-induced fold mRNA expression of indicated genes. **: P<0.01, compared to the conditions indicated (m = 3). **B:** mRNA expressions of known glucocorticoid-responsive genes in the morning and the evening. Relative mRNA expressions of *annexin A1*, *GILZ*, *IFNγ* and *IL-12 p40* at 8 am (Day) and 8 pm (Night) in PMBCs obtained from 10 healthy subjects are shown. Bars represent mean ± S.E. values of relative mRNA expression of the genes indicated. **: P<0.01, n.s.: not significant, compared to the conditions indicated (n = 10, m = 20).
(TIF)

Figure S2 Time-dependent alteration of hydrocortisone-stimulated *annexin A1* mRNA expression *ex vivo*. PBMCs obtained at 6 am from 6 healthy subjects were cultured in the medium and were treated with 5×10^−7^ M of hydrocortisone (HC) for 3 hours at every 6 hours. mRNA levels of *annexin A1* were then determined. Experiments were performed with duplicate in each subject. Circles represent the mean ± S.E. values of hydrocortisone (HC)-induced fold mRNA expression of *annexin A1*. Values obtained in the absence of hydrocortisone (HC) at time “0” were employed as a control **: P<0.01, compared to the values obtained at time “0” in the presence of hydrocortisone (n = 6, m = 12).
(TIF)

Figure S3 Knockdown of *Clock* mRNA abolishes diurnal fluctuation of *annexin A1* mRNA expression in PBMCs cultured *ex vivo*. PBMCs obtained at 6 am from 3 healthy subjects were transfected with Clock or control siRNA and were treated with 5×10^−7^ M of hydrocortisone (HC) for 3 hours at every 6 hours. mRNA expression of *annexin A1* was determined. Experiments were performed with duplicate for each subject. Circles represent the mean ± S.E. values of fold mRNA expression of the indicated genes obtained in the absence and presence of hydrocortisone (HC). The values obtained in the absence of hydrocortisone (HC) were employed as controls. **: P<0.01, n.s.: not significant, compared to the values obtained in the presence of hydrocortisone (HC) at time “0” for mRNA expression of glucocorticoid-responsive genes (n = 3, m = 6).
(TIF)
