# Supplementary figures and images for: Peripheral CLOCK Regulates Target-Tissue Glucocorticoid Receptor Transcriptional Activity in a Circadian Fashion in Man
Source: PLoS One. 2011 Sep 28;6(9):e25612. doi: 10.1371/journal.pone.0025612 (PMC3182238; doi:10.1371/journal.pone.0025612)

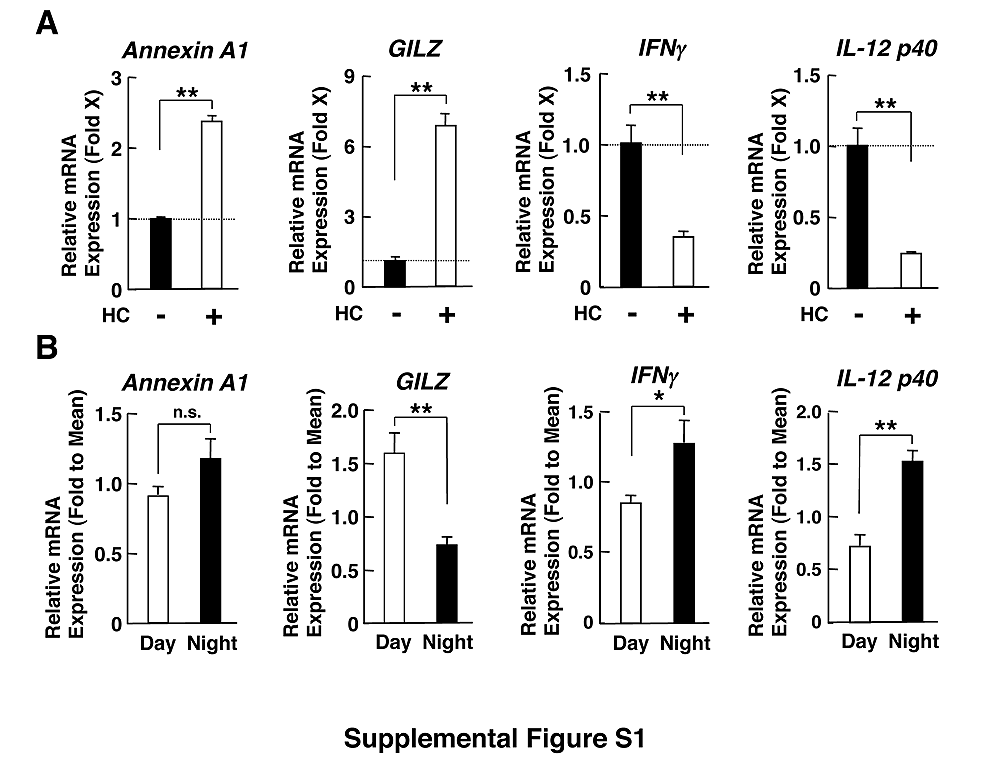

Supplement: Figure S1 — Response of glucocorticoid-responsive gene mRNA expressions to hydrocortisone in EBV-transformed peripheral lymphocytes and their daily changes in PBMCs. A: The effect of hydrocortisone on the expression of the mRNAs of known glucocorticoid-responsive genes in EBV-transformed peripheral lymphocytes. Samples obtained as in Figure 3 were used for the evaluation of the mRNA expressions of the known glucocorticoid-responsive genes indicated. Annexin A1 and GILZ are known to be up-regulated by glucocorticoids, while IFNγ and IL-12 p40 are known to be down-regulated. Bars represent the mean ± S.E. values of hydrocortisone (HC)-induced fold mRNA expression of indicated genes. **: P<0.01, compared to the conditions indicated (m = 3). B: mRNA expressions of known glucocorticoid-responsive genes in the morning and the evening. Relative mRNA expressions of annexin A1, GILZ, IFNγ and IL-12 p40 at 8 am (Day) and 8 pm (Night) in PMBCs obtained from 10 healthy subjects are shown. Bars represent mean ± S.E. values of relative mRNA expression of the genes indicated. **: P<0.01, n.s.: not significant, compared to the conditions indicated (n = 10, m = 20). (TIF) [file pone.0025612.s001.tif]

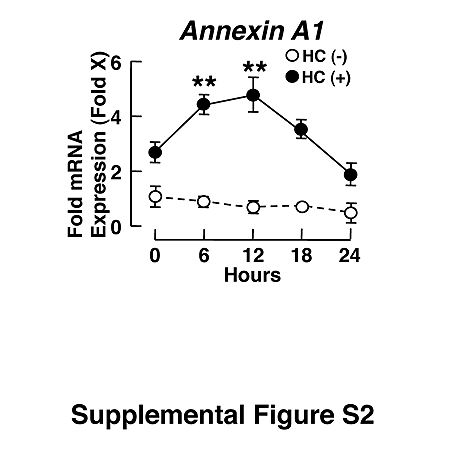

Supplement: Figure S2 — Time-dependent alteration of hydrocortisone-stimulated annexin A1 mRNA expression ex vivo. PBMCs obtained at 6 am from 6 healthy subjects were cultured in the medium and were treated with 5×10^−7^ M of hydrocortisone (HC) for 3 hours at every 6 hours. mRNA levels of annexin A1 were then determined. Experiments were performed with duplicate in each subject. Circles represent the mean ± S.E. values of hydrocortisone (HC)-induced fold mRNA expression of annexin A1. Values obtained in the absence of hydrocortisone (HC) at time “0” were employed as a control **: P<0.01, compared to the values obtained at time “0” in the presence of hydrocortisone (n = 6, m = 12). (TIF) [file pone.0025612.s002.tif]

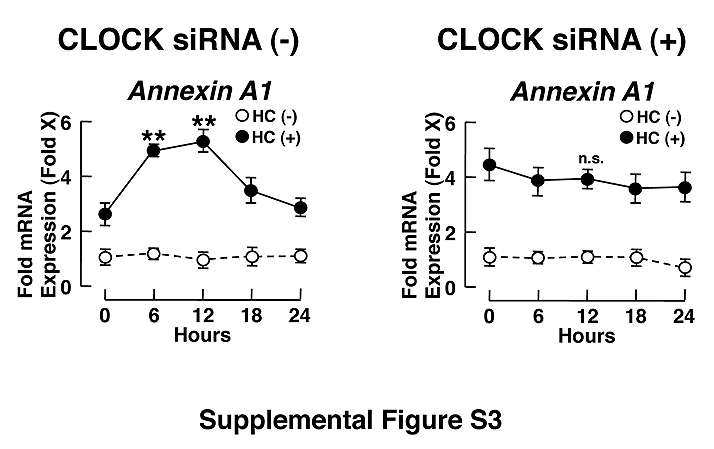

Supplement: Figure S3 — Knockdown of Clock mRNA abolishes diurnal fluctuation of annexin A1 mRNA expression in PBMCs cultured ex vivo. PBMCs obtained at 6 am from 3 healthy subjects were transfected with Clock or control siRNA and were treated with 5×10^−7^ M of hydrocortisone (HC) for 3 hours at every 6 hours. mRNA expression of annexin A1 was determined. Experiments were performed with duplicate for each subject. Circles represent the mean ± S.E. values of fold mRNA expression of the indicated genes obtained in the absence and presence of hydrocortisone (HC). The values obtained in the absence of hydrocortisone (HC) were employed as controls. **: P<0.01, n.s.: not significant, compared to the values obtained in the presence of hydrocortisone (HC) at time “0” for mRNA expression of glucocorticoid-responsive genes (n = 3, m = 6). (TIF) [file pone.0025612.s003.tif]
